# Supplementary material for: Out of Asia: mitochondrial evolutionary history of the globally introduced supralittoral isopod Ligia exotica
Source: PeerJ. 2018 Mar 12;6:e4337. doi: 10.7717/peerj.4337 (PMC5853605; doi:10.7717/peerj.4337)
Supplement: Supplemental Information 1 — Table S1. Information on the samples included in this study. ID labels correspond with those used in the figures. Table S2. Settings for Maximum Likelihood and Bayesian analyses. Â All others default; Bˆ Average Standard Deviation of Split Frequencies; Ĉ estimated in Tracer v.1.6; Dˆ Effective Sample Size; Ê Potential Scale Reduction Factor for all parameters. Table S3. A. Support for the clades defined in Fig. 2 from different methods and substitution models for the 16S rDNA dataset. B. Support for the clades defined in Fig. 2 from different methods and substitution models for the 16S rDNA and 12S rDNA concatenated dataset. n/a = not applicable (i.e., relationship could not be examined with this dataset). [file peerj-06-4337-s001.docx]

**TABLE S1**

Information on the samples included in this study. ID labels correspond with those used in the figures.

| **Species** | **Locality Names** | **ID** | **16S rDNA Accession Numbers** | **Nak** | **12S** | **Sources** | **Lat** | **Long** |
| --- | --- | --- | --- | --- | --- | --- | --- | --- |
| *L. exotica* | Goodland, FL, USA | 1 | KX447715 |  |  | This study | 25°55'57''N | 81°39'21''W |
| *L. exotica* | Sunshine Skyway Bridge North Rest Area, St. Petersburg, FL, USA | 2 | KX447719 |  |  | This study | 27°39'14''N | 82°40'41''W |
| *L. exotica* | Cedar Key, FL, USA | 3 | KX447717 |  |  | This study | 29°8'8''N | 83°2'11''W |
| *L. exotica* | Eastpoint, FL, USA | 4 | KX447734 |  |  | This study | 29°44'21''N | 84°52'25''W |
| *L. exotica* | Pensacola, FL, USA | 5 | KX447722 | MG676441 |  | This study | 30°25'11''N | 87°11'36''W |
| *L. exotica* | Biloxi Small Craft Harbor, Biloxi, MS, USA | 6 | KX447718 |  |  | This study | 30°23'31''N | 88°53'8''W |
| *L. exotica* | Long Beach Harbor, Biloxi, MS, USA | 7 | KX447721 |  |  | This study | 30°20'41''N | 89°8'42''W |
| *L. exotica* | Avery Island, LA, USA | 8 | KX447730 |  |  | This study | 29°54'57''N | 91°54'14''W |
| *L. exotica* | Galveston, TX, USA | 9 | Identical to KX447730 |  |  | This study | N/A | N/A |
| *L. exotica* | Palacios, TX, USA | 10 | KX447716 |  |  | This study | 28°44'18''N | 96°24'6''W |
| *L. exotica* | Municipal Harbor, Port Aransas, TX, USA | 11 | KX447720 | MG676436 | MG676421 | This study | 27°50'24''N | 97°3'50''W |
| *L. exotica* | South Padre Island, TX, USA | 12 | KX447723 |  |  | This study | 26°4'44''N | 97°10'9''W |
| *L. exotica* | San Juan de Ulúa Fort, Veracruz, Mexico | 13 | KF546552 |  | MG676420 | Santamaria *et al.* 2013 and this study | 19°12'34''N | 96°7'51''W |
| *L. exotica* | Jetty by Adolfo Ruiz Cortines statue, Veracruz, Mexico | 14 | KX447731 | MG676434 | MG676419 | This study | 19°11'40''N | 96°7'24''W |
| *L. exotica* | Cumberland Island, GA, USA | 15 | AF260861 |  |  | Wetzer 2001 | 30°51'N | 81°27'W |
| *L. exotica* | Chaguaramas Bay, Trinidad, Trinidad and Tobago | 16 | KX447732 |  |  | This study | 10°40'57''N | 61°37'21''W |
| *L. exotica* | Chaguaramas Bay, Trinidad, Trinidad and Tobago | 17 | KX447733 |  |  | This study | 10°40'57''N | 61°37'21''W |
| *L. exotica* | Praia de Calhetas, Cabo de Santo Agostinho, Brazil | 18 | KX447725 |  | MG676413 | This study | 8°20'38''S | 34°56'43''W |
| *L. exotica* | Praia do Paraíso, Pernambuco, Brazil | 18A | Identical to KX447725 |  |  | This study | 8°21'S | 34°57’W |
| *L. exotica* | Rio de Janeiro, Brazil | 19A | Identical to KX447725 |  |  | This study | N/A | N/A |
| *L. exotica* | Lagoa Azul, Ilha Grande, Costa Verde, Brazil | 19 | KX447726 |  |  | This study | 23°11'S | 44°18'W |
| *L. exotica* | Hilo Harbor, Hawai’i, HI, USA | 20 | KX447728 |  | MG676416 | This study | 19°43'57''N | 155°3'26''W |
| *L. exotica* | Pearl Harbor, O’ahu, HI, USA | 21 | KX447729 |  | LexOah1 | This study | 21°21'50''N | 157°57'37''W |
| *L. exotica* | Honolulu Harbor, O'ahu, HI, USA | 22 | AY051339 |  |  | Taiti et al. 2003 | N/A | N/A |
| *L. exotica* | Vilankulos, Mozambique | 23 | KX447736 |  | MG676423 | This study | 21°59'52''S | 35°19'30''E |
| *L. exotica* | Beira, Mozambique | 24 | KX447737 |  | MG676422 | This study | 19°50'53''S | 34°53'35''E |
| *L. exotica* | Durban Harbor, KwaZulu-Natal, South Africa | 25 | KX447749 |  |  | This study | N/A | N/A |
| *L. exotica* | Blue Lagoon, Umgeni River Mouth, KwaZulu-Natal, South Africa | 26 | KX447750 |  |  | This study | 29°48'36''S | 31°2'8''E |
| *L. exotica* | Niigata, Japan | 27 | KX447747 | MG676439 | MG676410 | This study | 37°54'58''N | 139°2'11''E |
| *L. exotica* | Kanagawa, Japan | 28 | KX447727 | MG676443 | MG676405 | This study | 35°9'25''N | 139°36'43''E |
| *L. exotica* | Toyohashi, Japan | 29 | KX447741 | MG676435 | MG676417 | This study | N/A | N/A |
| *L. exotica* | Fukuoka, Japan | 30 | KX447746 | MG676430 | MG676408 | This study | 33°35'N | 130°24'E |
| *L. cinerascens* | Otaru, Japan | NA | KX447751 | MG676428 | MG676402 | This study | 43°11'N | 141°E |
| *L. cinerascens* | Sendai, Japan | NA | KX447753 | MG676427 | MG676400 | This study | 38°16'N | 140°52'E |
| *L. exotica* | Kitadaito son, Okinawa, Japan | 31 | KX447738 |  | MG676411 | This study | 25°56'45''N | 131°17'56''E |
| *L. exotica* | Okinawa, Japan | 32 | KX447742 | MG676429 | MG676412 | This study | 26°28'46''N | 127°55'40''E |
| *Ligia sp.* | Okinawa, Japan | 33 | AY606110 |  |  | GenBank | N/A | N/A |
| *Ligia sp.* | Okinawa, Japan | 34 | AY606109 |  |  | GenBank | N/A | N/A |
| *Ligia sp.* | Okinawa, Japan | 35 | AY606108 |  |  | GenBank | N/A | N/A |
| *Ligia sp.* | Okinawa, Japan | 36 | AY606107 |  |  | GenBank | N/A | N/A |
| *Ligia sp.* | Okinawa, Japan | 37 | AY606106 |  |  | GenBank | N/A | N/A |
| *L. exotica* | Ulleungdo Island, South Korea | 38 | KX447743 |  | MG676409 | This study | 37°30'6''N | 130°51'11''E |
| *L. exotica* | Boryeong, South Korea | 39 | KX447744 | MG676433 | MG676406 | This study | 38°4'53''N | 127°38'16''E |
| *L. exotica* | Woojuk, South Korea | 40 | KX447745 | MG676432 |  | This study | N/A | N/A |
| *L. cinerascens* | Boryeong, South Korea | NA | KX447754 |  | MG676403 | This study | 38°4'53''N | 127°38'16''E |
| *L. cinerascens* | Boseong, South Korea | NA | KX447755 | MG676426 | MG676401 | This study | 34°48'28''N | 127°8'15''E |
| *L. cinerascens* | Cheju, South Korea | NA | KX447756 | MG676425 | MG676404 | This study | 33°22'N | 126°32'E |
| *Ligia sp.* | Western Group, South Korea | NA | AY545635 |  |  | Jung *et al*. 2008 | 36°48'44''N | 126°18'37''E |
| *Ligia sp.* | Western Group, South Korea | NA | AY545634 |  |  | Jung *et al*. 2008 | 35°05'40''N | 126°27'42''E |
| *Ligia sp.* | Western Group, South Korea | NA | AY545633 |  |  | Jung *et al*. 2008 | 37°26'37''N | 126°22'38''E |
| *Ligia sp.* | Western Group, South Korea | NA | AY545632 |  |  | Jung *et al*. 2008 | 36°09'32''N | 126°29'34''E |
| *Ligia sp.* | Western Group, South Korea | NA | AY545631 |  |  | Jung *et al*. 2008 | 37°17'04''N | 126°29'34''E |
| *Ligia sp.* | Western Group, South Korea | NA | AY545630 |  |  | Jung *et al*. 2008 | 35°05'40''N | 126°27'42''E |
| *Ligia sp.* | Western Group, South Korea | NA | AY545629 |  |  | Jung *et al*. 2008 | N/A | N/A |
| *Ligia sp.* | Western Group, South Korea | NA | AY545628 |  |  | Jung *et al*. 2008 | 37°35'41''N | 126°26'46''E |
| *Ligia sp.* | Western Group, South Korea | NA | AY545627 |  |  | Jung *et al*. 2008 | 36°48'44''N | 126°18'37''E |
| *Ligia sp.* | Western Group, South Korea | NA | AY545626 |  |  | Jung *et al*. 2008 | 34°40'47''N | 127°06'34''E |
| *Ligia sp.* | Western Group, South Korea | NA | AY545625 |  |  | Jung *et al*. 2008 | 36°48'44''N | 126°18'37''E |
| *Ligia sp.* | Western Group, South Korea | NA | AY545624 |  |  | Jung *et al*. 2008 | N/A | N/A |
| *Ligia sp.* | Western Group, South Korea | NA | AY545623 |  |  | Jung *et al*. 2008 | 35°06'12''N | 128°29'54''E |
| *Ligia sp.* | Western Group, South Korea | NA | AY545622 |  |  | Jung *et al*. 2008 | 37°35'41''N | 126°26'46''E |
| *Ligia sp.* | Western Group, South Korea | NA | AY545621 |  |  | Jung *et al*. 2008 | 36°09'32''N | 126°34'09''E |
| *Ligia sp.* | Western Group, South Korea | NA | AY545620 |  |  | Jung *et al*. 2008 | 37°17'04''N | 126°29'34''E |
| *Ligia sp.* | Western Group, South Korea | NA | AY545619 |  |  | Jung *et al*. 2008 | 37°17'04''N | 126°29'34''E |
| *Ligia sp.* | Western Group, South Korea | NA | AY545618 |  |  | Jung *et al*. 2008 | 36°09'32''N | 126°29'34''E |
| *Ligia sp.* | Western Group, South Korea | NA | AY545617 |  |  | Jung *et al*. 2008 | 35°05'40''N | 126°27'42''E |
| *Ligia sp.* | Western Group, South Korea | NA | AY545616 |  |  | Jung *et al*. 2008 | 35°06'12''N | 128°29'54''E |
| *Ligia sp.* | Western Group, South Korea | NA | AY545615 |  |  | Jung *et al*. 2008 | N/A | N/A |
| *Ligia sp.* | Western Group, South Korea | NA | AY545614 |  |  | Jung *et al*. 2008 | N/A | N/A |
| *Ligia sp.* | Eastern Group, South Korea | 41 | EU213044 |  |  | Jung *et al*. 2008 | 37°29'07''N | 130°54'23''E |
| *Ligia sp.* | Eastern Group, South Korea | 42 | EU213043 |  |  | Jung *et al*. 2008 | 37°29'07''N | 130°54'23''E |
| *Ligia sp.* | Eastern Group, South Korea | 43 | EU213042 |  |  | Jung *et al*. 2008 | 37°29'07''N | 130°54'23''E |
| *Ligia sp.* | Eastern Group, South Korea | 44 | AY545613 |  |  | Jung *et al*. 2008 | 35°42'17''N | 126°35'19''E |
| *Ligia sp.* | Eastern Group, South Korea | 45 | AY545612 |  |  | Jung *et al*. 2008 | 33°13'04''N | 126°30'49''E |
| *Ligia sp.* | Eastern Group, South Korea | 46 | AY545611 |  |  | Jung *et al*. 2008 | 34°57'09''N | 127°46'40''E |
| *Ligia sp.* | Eastern Group, South Korea | 47 | AY545610 |  |  | Jung *et al*. 2008 | 37°20'43''N | 129°15'46''E |
| *Ligia sp.* | Eastern Group, South Korea | 48 | AY545609 |  |  | Jung *et al*. 2008 | 35°13'05''N | 129°14'03''E |
| *Ligia sp.* | Eastern Group, South Korea | 49 | AY545608 |  |  | Jung *et al*. 2008 | 35°13'05''N | 129°14'03''E |
| *Ligia sp.* | Eastern Group, South Korea | 50 | AY545607 |  |  | Jung *et al*. 2008 | 34°57'09''N | 127°46'40''E |
| *Ligia sp.* | Eastern Group, South Korea | 51 | AY545606 |  |  | Jung *et al*. 2008 | 36°59'15''N | 129°25'04''E |
| *Ligia sp.* | Eastern Group, South Korea | 52 | AY545605 |  |  | Jung *et al*. 2008 | N/A | N/A |
| *Ligia sp.* | Eastern Group, South Korea | 53 | AY545604 |  |  | Jung *et al*. 2008 | 34°57'09''N | 127°46'40''E |
| *Ligia sp.* | Eastern Group, South Korea | 54 | AY545603 |  |  | Jung *et al*. 2008 | 35°13'05''N | 129°14'03''E |
| *Ligia sp.* | Eastern Group, South Korea | 55 | AY545602 |  |  | Jung *et al*. 2008 | 35°13'05''N | 129°14'03''E |
| *Ligia sp.* | Eastern Group, South Korea | 56 | AY545601 |  |  | Jung *et al*. 2008 | N/A | N/A |
| *Ligia sp.* | Eastern Group, South Korea | 57 | AY545600 |  |  | Jung *et al*. 2008 | 34°40'47''N | 127°06'34''E |
| *L. exotica* | Lutao, Taitung, Taiwan | 58 | KX447735 | MG676438 | MG676414 | This study | N/A | N/A |
| *L. exotica* | Pingtung County, Taiwan | 59 | KX447740 |  |  | This study | 22°29'44''N | 120°36'52''E |
| *Ligia sp.* | Chinmen Tao, Taiwan | 60 | AY606099 |  |  | GenBank | N/A | N/A |
| *Ligia sp.* | Chinmen Tao, Taiwan | 61 | AY606098 |  |  | GenBank | N/A | N/A |
| *Ligia sp.* | Chinmen Tao, Taiwan | 62 | AY606097 |  |  | GenBank | N/A | N/A |
| *Ligia sp.* | Chinmen Tao, Taiwan | 63 | AY606096 |  |  | GenBank | N/A | N/A |
| *Ligia sp.* | Chinmen Tao, Taiwan | 64 | AY606095 |  |  | GenBank | N/A | N/A |
| *Ligia sp.* | Chinmen Tao, Taiwan | 65 | AY606094 |  |  | GenBank | N/A | N/A |
| *Ligia sp.* | Chinmen Tao, Taiwan | 66 | AY606093 |  |  | GenBank | N/A | N/A |
| *Ligia sp.* | Chilung, Taiwan | 67 | AY606105 |  |  | GenBank | N/A | N/A |
| *Ligia sp.* | Chilung, Taiwan | 68 | AY606104 |  |  | GenBank | N/A | N/A |
| *Ligia sp.* | Chilung, Taiwan | 69 | AY606103 |  |  | GenBank | N/A | N/A |
| *Ligia sp.* | Chilung, Taiwan | 70 | AY606102 |  |  | GenBank | N/A | N/A |
| *Ligia sp.* | HuapingHsu, Taiwan | 71 | AY606101 |  |  | GenBank | N/A | N/A |
| *Ligia sp.* | HuapingHsu, Taiwan | 72 | AY606100 |  |  | GenBank | N/A | N/A |
| *L. exotica* | Tianjin and Shandong, China | 73 | JX414122 |  |  | Yin *et al*. 2013 | N/A | N/A |
| *L. exotica* | Rushan, Shandong, China | 74 | JX414123 |  |  | Yin *et al*. 2013 | 36°50'59''N | 121°36'50''E |
| *L. exotica* | Rushan, Shandong, China | 75 | JX414124 |  |  | Yin *et al*. 2013 | 36°50'59''N | 121°36'50''E |
| *L. exotica* | Rushan, Shandong, China | 76 | JX414125 |  |  | Yin *et al*. 2013 | 36°50'59''N | 121°36'50''E |
| *L. exotica* | Shandong, China | 77 | JX414126 |  |  | Yin *et al*. 2013 | N/A | N/A |
| *L. exotica* | Weihai, Shandong, China | 78 | JX414127 |  |  | Yin *et al*. 2013 | 37°26'14''N | 122°9'42''E |
| *L. exotica* | Qingdao-Zhanqiao, Shandong, China | 79 | JX414128 |  |  | Yin *et al*. 2013 | 36°3'41''N | 120°19'10''E |
| *L. exotica* | Qingdao-Zhanqiao, Shandong, China | 80 | JX414129 |  |  | Yin *et al*. 2013 | 36°3'41''N | 120°19'10''E |
| *L. exotica* | Qingdao-Zhanqiao, Shandong, China | 81 | JX414130 |  |  | Yin *et al*. 2013 | 36°3'41''N | 120°19'10''E |
| *L. exotica* | Qingdao-Hongdao, Shandong, China | 82 | JX414131 |  |  | Yin *et al*. 2013 | 36°10'58''N | 120°16'57''E |
| *L. exotica* | Qingdao-Hongdao, Shandong, China | 83 | JX414132 |  |  | Yin *et al*. 2013 | 36°10'58''N | 120°16'57''E |
| *L. exotica* | Qingdao-Hongdao, Shandong, China | 84 | JX414133 |  |  | Yin *et al*. 2013 | 36°10'58''N | 120°16'57''E |
| *L. exotica* | Qingdao, Shandong, China | 85 | KX447748 | MG676431 | MG676407 | This study | 36°3'58''N | 120°22'10''E |
| *L. exotica* | Zhujiajian Island, Zhejiang, China | 86 | KX447739 | MG676440 | MG676418 | This study | 29°54''N | 122°53'E |
| *L. exotica* | Lianyungang, Jiangsu, China | 87 | JX414134 |  |  | Yin *et al*. 2013 | 34°46'32''N | 119°26'34''E |
| *L. exotica* | Jiangsu, China | 88 | JX414135 |  |  | Yin *et al*. 2013 | N/A | N/A |
| *L. exotica* | Nantong, Jiangsu, China | 89 | JX414136 |  |  | Yin *et al*. 2013 | 32°5'7''N | 121°35'51''E |
| *L. exotica* | Nantong, Jiangsu, China | 90 | JX414137 |  |  | Yin *et al*. 2013 | 32°5'7''N | 121°35'51''E |
| *L. exotica* | Nantong, Jiangsu, China | 91 | JX414138 |  |  | Yin *et al*. 2013 | 32°5'7''N | 121°35'51''E |
| *L. exotica* | Zhujiazian, Zhoushan Islands, China | 92 | KJ802850 |  |  | GenBank | 29°52′12″N | 122°23′55″E |
| *L. exotica* | Zhujiazian, Zhoushan Islands, China | 93 | KJ802851 |  |  | GenBank | 29°52′12″N | 122°23′55″E |
| *L. exotica* | Zhujiazian, Zhoushan Islands, China | 94 | KJ802852 |  |  | GenBank | 29°52′12″N | 122°23′55″E |
| *L. exotica* | Zhujiazian, Zhoushan Islands, China | 95 | KJ802853 |  |  | GenBank | 29°52′12″N | 122°23′55″E |
| *L. exotica* | Zhujiazian, Zhoushan Islands, China | 96 | KJ802854 |  |  | GenBank | 29°52′12″N | 122°23′55″E |
| *L. cinerascens* | Tianjin, China | NA | KX447752 | TJ1 |  | This study | 39°08'N | 117°11'E |
| *L. cinerascens* | Northeast and East China | NA | JX414115 |  |  | Yin *et al*. 2013 | N/A | N/A |
| *L. cinerascens* | Liaoning, China | NA | JX414116 |  |  | Yin *et al*. 2013 | N/A | N/A |
| *L. cinerascens* | Liaoning and Shandong, China | NA | JX414117 |  |  | Yin *et al*. 2013 | N/A | N/A |
| *L. cinerascens* | Huludao, Liaoning, China | NA | JX414118 |  |  | Yin *et al*. 2013 | 40°40'30''N | 120°49'33''E |
| *L. cinerascens* | Tianjin and Shandong, China | NA | JX414119 |  |  | Yin *et al*. 2013 | N/A | N/A |
| *L. cinerascens* | Liaoning and Shandong, China | NA | JX414120 |  |  | Yin *et al*. 2013 | N/A | N/A |
| *L. cinerascens* | Dalian-Heishijiao, Liaoning, China | NA | JX414121 |  |  | Yin *et al*. 2013 | 38°57'53''N | 121°18'53''E |
| *Ligia sp.* | Cambodia | 97 | AY606092 |  |  | GenBank | N/A | N/A |
| *Ligia sp.* | Cambodia | 98 | AY606091 |  |  | GenBank | N/A | N/A |
| *Ligia sp.* | Cambodia | 99 | AY606090 |  |  | GenBank | N/A | N/A |
| *Ligia sp.* | Cambodia | 100 | AY606089 |  |  | GenBank | N/A | N/A |
| *Ligia sp.* | Cambodia | 101 | AY606088 |  |  | GenBank | N/A | N/A |
| *Ligia sp.* | Cambodia | 102 | AY606087 |  |  | GenBank | N/A | N/A |
| *Ligia sp.* | Cambodia | 103 | AY606086 |  |  | GenBank | N/A | N/A |
| *L. exotica* | Parangipetta, India | 104 | KX447724 | MG676437 |  | This study | 11°29'24''N | 79°45'36''E |
| *L. exotica* | Punta Carretas, Montevideo, Uruguay | 105A | Identical to AY606090 |  |  | This study | 34°56'06'' S | 56°09'40'' W |
| *L. exotica* | Punta Carretas, Montevideo, Uruguay | 105B | Identical to AY606101 |  |  | This study | 34°56'06'' S | 56°09'40'' W |
| *L. exotica* | Orchid Island, Taiwan | 106 |  |  | Identical to MG676419 and MG676420 | This study | 22°04'51"N | 121°30'44"E |
|  |  |  |  |  |  |  |  |  |

**TABLE S2**

Settings for Maximum Likelihood and Bayesian analyses. ^A^ All others default; ^B^ Average Standard Deviation of Split Frequencies; ^C^ estimated in Tracer v.1.6; ^D^ Effective Sample Size; ^E^ Potential Scale Reduction Factor for all parameters.

| Method | Model and Priors ^A^ | Iterations Generations / Bootstrap Replicates | Sample Frequency | Runs/Chains | Burn-in | ASDSF ^B^ | Bayes Factors / ML Scores (-lLn) ^C^ | ESS>200 ^D^ | PSRF ^E^ |
| --- | --- | --- | --- | --- | --- | --- | --- | --- | --- |
| RAxML | GTR+Γ | 1,000 | n/a | n/a | n/a | n/a | -1936.5887 | n/a | n/a |
| RAxML | HKY+I+Γ | 1,000 | n/a | n/a | n/a | n/a | -1946.8587 | n/a | n/a |
| GARLI | GTR+Γ | 1,000 | n/a | n/a | n/a | n/a | -1802.5711 | n/a | n/a |
| GARLI | TPM2uf+I+Γ | 1,000 | n/a | n/a | n/a | n/a | -2055.0212 | n/a | n/a |
| GARLI | HKY+I+Γ | 1,000 | n/a | n/a | n/a | n/a | -1650.6554 | n/a | n/a |
| PhyML | GTR+Γ | 1,000 | n/a | n/a | n/a | n/a | n/a | n/a | n/a |
| PhyML | HKY+I+Γ | 1,000 | n/a | n/a | n/a | n/a | n/a | n/a | n/a |
| MrBayes | GTR+Γ | 100,000,000 | 10,000 | 4 | 10% | 0.003743 | -2213.7510 | Yes | 1 |
| MrBayes | TPM2uf+I+Γ | 100,000,000 | 10,000 | 4 | 10% | 0.003915 | -2218.5443 | Yes | 1 |
| MrBayes | HKY+I+Γ | 100,000,000 | 10,000 | 4 | 10% | 0.003395 | -2217.5341 | Yes | 1 |
| Phycas | GTR+Γ | 1,000,000 | 10 | 1/1 | 10% | n/a | -2193.3224 | Yes | n/a |
| Phycas | HKY+I+Γ | 1,000,000 | 10 | 1/1 | 10% | n/a | -2201.1564 | Yes | n/a |

**TABLE S3**

A. Support for the clades defined in Figure 2 from different methods and substitution models for the 16S rDNA dataset

| Clade | RAxML - GTR+Γ | GARLI - GTR+Γ | GARLI - TPM2uf+I+Γ | GARLI - HKY+I+Γ | PhyML - GTR+Γ | PhyML - HKY+I+Γ | MrBayes - GTR+Γ | MrBayes - TPM2uf+I+Γ | MrBayes - HKY+I+Γ | Phycas - GTR+Γ | Phycas - HKY+I+Γ |
| --- | --- | --- | --- | --- | --- | --- | --- | --- | --- | --- | --- |
| B | 81 | 82 | 80 | 62 | 92 | 87 | 100 | 100 | 100 | 100 | 100 |
| C | 78 | 74 | 60 | 74 | 79 | 75 | 96 | 95 | 97 | 99 | 100 |
| D | 88 | 80 | 64 | 76 | 93 | 87 | 100 | 100 | 99 | 100 | 100 |
| D1 | 50 | <50 | 64 | <50 | 57 | 58 | 90 | 87 | 83 | 95 | 85 |
| D2 | 99 | 98 | 84 | 100 | 99 | 98 | 100 | 100 | 100 | 100 | 100 |
| D3 | 60 | 56 | 68 | <50 | 82 | 77 | 83 | 72 | 69 | 86 | 68 |
| D2+D3 | 64 | 60 | <50 | <50 | 57 | 65 | 60 | 56 | 62 | 60 | 76 |
| C+D | 70 | 60 | <50 | <50 | 65 | 71 | 82 | 94 | 77 | 99 | 99 |
| B+C+D | 77 | 76 | 74 | <50 | 74 | 67 | 100 | 100 | 100 | 100 | 100 |
| *L. exotica* clade vs.  *L. cinerascens* split | 100 | 99 | 98 | 99 | 100 | 100 | 100 | 100 | 100 | 100 | 100 |
|  |  |  |  |  |  |  |  |  |  |  |  |

B. Support for the clades defined in Figure 2 from different methods and substitution models for the 16S rDNA and 12S rDNA concatenated dataset. n/a = not applicable (i.e., relationship could not be examined with this dataset).

| Clade | RAxML - GTR+Γ | GARLI - GTR+Γ | GARLI - TPM2uf+I+Γ | GARLI - HKY+I+Γ | PhyML - GTR+Γ | PhyML - HKY+I+Γ | MrBayes - GTR+Γ | MrBayes - TPM2uf+I+Γ | MrBayes - HKY+I+Γ |
| --- | --- | --- | --- | --- | --- | --- | --- | --- | --- |
| B | 100 | 100 | 100 | 100 | 100 | 100 | 100 | 100 | 100 |
| C | n/a | n/a | n/a | n/a | n/a | n/a | n/a | n/a | n/a |
| D | 82 | 82 | 78 | 76 | 93 | 85 | 99 | 98 | 99 |
| D1 | n/a | n/a | n/a | n/a | n/a | n/a | n/a | n/a | n/a |
| D2 | 100 | 100 | 100 | 100 | 98 | 100 | 100 | 100 | 100 |
| D3 | 59 | <50 | <50 | <50 | 53 | <50 | <50 | <50 | <50 |
| D2+D3 | 71 | 74 | 73 | 70 | 73 | 61 | 60 | 94 | 95 |
| C+D | 94 | 90 | 89 | 86 | 94 | 92 | 100 | 100 | 100 |
| B+C+D | 98 | 100 | 100 | 100 | 100 | 98 | 100 | 100 | 100 |
| *L. exotica* clade vs.  *L. cinerascens* split | 100 | 99 | 98 | 99 | 100 | 100 | 100 | 100 | 100 |
|  |  |  |  |  |  |  |  |  |  |
